# Effectiveness of a multi-surgical department online recruitment event ‘GEKA-Navi’ on career motivation and residency entry: a 5-year experience

**DOI:** 10.1007/s00595-026-03362-5

**Published:** 2026-06-18

**Authors:** Shota Nakamura, Hideki Takami, Shunsuke Onoe, Taisuke Baba, Taro Fujii, Satoshi Makita, Yuki Masano, Shuta Ikeda, Dai Takeuchi, Toyofumi Fengshi Chen-Yoshikawa

**Affiliations:** 1 Department of Thoracic Surgery, Nagoya University Graduate School of Medicine, 65 Tsurumai-cho, Showa-ku, Nagoya, 466-8550 Japan; 2 Department of Surgery, Nagoya University Graduate School of Medicine, Nagoya, Japan; 3 Department of Cardiac Surgery, Nagoya University Graduate School of Medicine, Nagoya, Japan; 4 Department of Pediatric Surgery, Nagoya University Graduate School of Medicine, Nagoya, Japan; 5 Department of Transplantation Surgery, Nagoya University Hospital, Nagoya, Japan; 6 Division of Vascular and Endovascular Surgery, Department of Surgery, Nagoya University Graduate School of Medicine, Nagoya, Japan; 7 Department of Breast and Endocrine Surgery, Nagoya University Graduate School of Medicine, Nagoya, Japan

**Keywords:** Surgical workforce, Surgical education, Early exposure, Career motivation, Residency recruitment, Recruitment strategy

## Abstract

**Purpose:**

This study aimed to evaluate the effectiveness of “GEKA-Navi,” a collaborative online recruitment event organized by eight surgical departments, and analyze the participant feedback and residency entry data from 2021 to 2025.

**Methods:**

GEKA-Navi targeted medical students and residents and featured plenary sessions on surgical careers, as well as departmental breakout sessions. The primary outcome was the proportion of newly recruited surgical residents from 2021 to 2025 who had participated in GEKA-Navi. The secondary outcomes were questionnaire-based participant evaluations, including satisfaction and motivation toward surgical careers.

**Results:**

Five events were conducted, with annual participation ranging from 82 to 150 individuals. A total of 151 new surgical residents entered the participating departments from 2021 to 2025, of whom 90 (59.6%) had previously attended GEKA-Navi. Secondary outcome analyses revealed consistently high participant satisfaction, with 98% of participants reporting increased interest in joining surgical departments and over 80% reporting increased motivation to become surgeons.

**Conclusions:**

GEKA-Navi was associated with high participant satisfaction and increased interest in surgical careers. These findings provide descriptive insights into participants’ perceptions and recruitment patterns within a structured multidepartmental recruitment initiative. Such approaches may help inform the design of future recruitment strategies for clinical trials.

## Introduction

In recent years, the decline in the number of applicants to surgical residency programs, together with nationwide workstyle reform and restrictions on clinical working hours, has raised concerns about securing the future surgical workforce in Japan [1, 2]. Similar issues have been reported in the United States, where declining interest among medical students in pursuing surgical careers has underscored the need to communicate more effectively the appeal and societal importance of surgery as a profession [3, 4].

Early exposure to surgical departments during medical school increases interest and fosters positive attitudes toward surgical careers. Such interventions are now considered crucial strategies for reversing the downward trend in surgical recruitment [5–7]. The shortage of young surgeons has become a critical concern at our institution and also in hospitals in the surrounding region. In 2021, we launched a multidepartment online event called GEKA-Navi. This annual program introduced the realities and attractiveness of surgical careers to medical students and surgical residents in an accessible and interactive manner.

In Japan, surgical training follows a structured pathway in which physicians complete a two-year postgraduate general clinical training program before entering general surgical training. Trainees select a subspecialty field after acquiring core competencies in general surgery. Each surgical department functions as an independent academic and administrative unit. However, collaboration across departments is common within affiliated and regional training hospitals, where general surgical education is frequently integrated. Within this context, a coordinated recruitment initiative involving multiple independent surgical departments holds particular institutional and educational significance. In contrast to conventional recruitment activities, which are typically conducted independently by each surgical department, GEKA-Navi represents an integrated, multidepartmental approach that encompasses eight surgical specialties. This structure enables the efficient aggregation of participants interested in surgical careers and facilitates comprehensive exposure to the diversity of surgical fields. Furthermore, the program is structured in a two-step format consisting of plenary sessions that highlight the overall appeal of surgical careers and small-group interactive sessions that enable an in-depth understanding of individual specialties. This combination provides a stepwise framework for career exploration, distinguishing it from traditional, one-directional, or single-session recruitment activities.

This study aimed to investigate the effectiveness of the 5-year initiative by analyzing participant feedback through post-event questionnaires and evaluating its association with actual residency entry. The results of this study provide timely and meaningful insights into novel approaches for surgical workforce development, when the decline in surgical manpower poses an urgent threat to healthcare delivery.

## Materials and methods

### Ethical consideration

A multi-surgical department online recruitment event, GEKA-Navi, was held at Nagoya University Hospital. This study was conducted as educational research and program evaluation to assess the perceptions of GEKA-Navi participants through a post-event questionnaire and investigate whether the event was associated with measurable outcomes in surgical residency recruitment. The study involved no medical intervention, clinical procedures, or alterations in standard educational or training practices. Questionnaire responses were collected anonymously and voluntarily, and no personally identifiable information was obtained from the participants. The study was conducted following the ethical principles of the Declaration of Helsinki, owing to the non-interventional nature of the study and the absence of individual-level identifiable data.

### Overview of GEKA-Navi

Historically, each surgical department at Nagoya University has independently conducted recruitment activities for surgical residency programs. However, a unified approach to surgical recruitment was initiated following the implementation of the new board certification system in Japan and the establishment of a common surgical training program across all departments. This helped develop a collaborative career guidance event called “GEKA-Navi” (short for GEKA Navigation), which brings together all surgical departments to conduct a joint residency information session. The eight participating departments were Gastrointestinal Surgery, Hepato-Biliary-Pancreatic Surgery, Thoracic Surgery, Cardiac Surgery, Vascular Surgery, Breast and Endocrine Surgery, Transplantation Surgery, and Pediatric Surgery.

In 2021, GEKA-Navi was launched and has since been held annually for five consecutive years as a fully online event using a web-based meeting platform. The event was originally planned as a large, in-person gathering; however, the onset of the coronavirus disease-2019 pandemic required a transition to an online format. This change ultimately enabled broader participation with reduced logistical constraints. The target audience includes junior and senior residents (postgraduate years 1–2 and 3–5) and medical students interested in surgical careers.

### The program of GEKA-Navi

Part I: Special Feature Sessions (30–60 min).

**Fig. 1:**
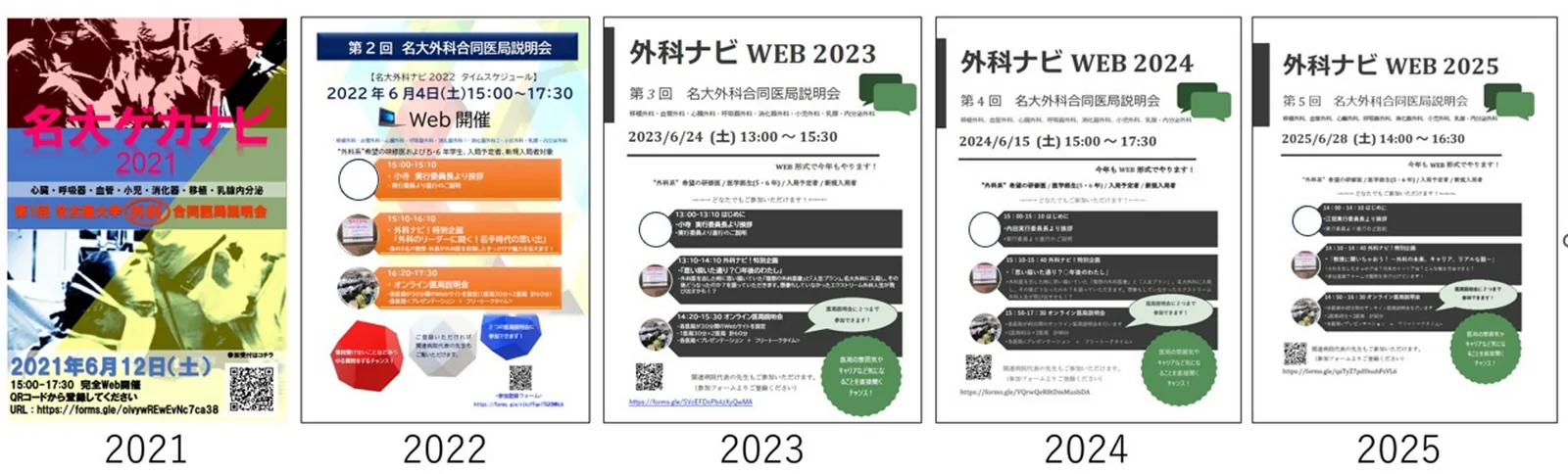
Poster of the GEKA-Navi program. Official promotional poster of GEKA-Navi used for annual recruitment. The poster highlights the program structure, including Part I (Special Feature Sessions with thematic lectures and discussions) and Part II (Departmental Breakout Sessions)

- A distinct theme is selected annually to provide participants with engaging insights into surgical life and career development (Fig. 1).

- During the inaugural session, all professors delivered motivational talks under the theme “Ask the Leaders: Why Choose Surgery?”
- In 2022, all professors delivered motivational talks under the theme “What Young Surgeons Can Learn from the Early Careers of Surgical Leaders?”
- In 2023 and 2024, young faculty members presented “What I Envisioned 10 years ago, Then, What I Became Now,” reflecting on how their early aspirations evolved after joining the department.
- In 2025, a roundtable discussion titled “let us Ask the Professors! Real Talk on the Future, Careers, and Lives of Surgeons” was held, during which students posed questions directly to professors.

Part II: Departmental Breakout Sessions (90 min).

After completing Part I, the participants attended two consecutive 45-minute departmental sessions of their choice. Each session provided detailed information regarding the department’s training environment, case volume, surgical style, academic activities, and work–life balance considerations. These small-group sessions enabled direct interaction with the faculty and current residents, thereby allowing personalized discussions.

### Participant recruitment

The target audience comprised medical students at all levels and junior residents of the specialty. Invitations and registration forms were distributed via email and student networks at Nagoya University and its affiliated institutions. Furthermore, the program was greatly promoted through the Internet and social media platforms. Participation was free of charge and voluntary.

### Evaluation and feedback

Immediately after each event, the participants were invited to complete an anonymous online questionnaire using Google Forms. The survey included quantitative and qualitative items covering the post-event questionnaire, which evaluated the following items:

- Overall satisfaction with Parts I and II of the program
- Perceived overall value of the program

- Appropriateness of the duration of each session

- Whether participation increased interest in pursuing a surgical career
- Whether participation increased interest in joining the surgical departments at Nagoya University
- Whether the program met the participants’ individual needs

- Willingness to participate in similar events in the future

Survey participation was voluntary and anonymous. The questionnaire structure remained largely consistent from 2021 to 2025, allowing for cross-year comparisons of key indicators.

### Outcomes

The primary outcome was the proportion of newly recruited surgical residents in the eight participating departments from 2021 to 2025 who had participated in GEKA-Navi. The secondary outcomes were questionnaire-based participant evaluations, including satisfaction with the program, motivation toward a surgical career, interest in joining the surgical departments at Nagoya University, perceived match between the program and participants’ needs, and willingness to participate in similar future events.

### Residency entry data and analysis

We reviewed the residency records of all eight surgical departments from 2021 to 2025 to assess the association between GEKA-Navi participation and actual residency entries. The number of new residents per year was identified, and their participation in GEKA-Navi before entry was monitored.

### Statistical analyses

Microsoft Excel (Microsoft Corporation, Redmond, WA, USA) was used for statistical analysis to compile the survey responses and generate graphical representations. Descriptive statistics were calculated for each survey question, and bar charts were created to visualize the response distributions.

## Results

### Participant demographics and survey response rate

Five GEKA-Navi events were held from 2021 to 2025, with the number of participants per year ranging from 82 to 150, comprising medical students, junior residents (postgraduate years 1–2), senior residents (postgraduate years 3–5), and young or attending surgeons (Table 1). A total of 111 participants attended the 2025 event, comprising 23 medical students (20.7%), 61 junior residents (55%), 7 senior residents (6.3%), and 20 young or attending surgeons (18.0%). Of these participants, 20 young or attending surgeons were excluded, leaving 91 participants, among whom 44 completed the questionnaire, leading to a response rate of 48.4%. Of the responses, 14 (31.8%) were from medical students, 25 (56.8%) from junior residents, and 5 (11.4%) from senior residents.

**Table 1:** Participant Demographics and Survey Response Rate

	2021	2022	2023	2024	2025
Number of total participants	150	82	85	86	111
Medical students	35 (23.3%)	15 (18.3%)	13 (15.3%)	16 (18.6%)	23 (20.7%)
Junior residents (postgraduate years 1-2)	74 (49.3%)	40 (26.6%)	44 (51.8%)	50 (58.1%)	61 (55.0%)
Senior residents (postgraduate years 3-5)	17 (11.3%)	7 (8.5%)	12 (14.1%)	3 (3.5%)	7 (6.3%)
Young or attending surgeons	24 (16.0%)	20 (24.4%)	16 (18.8%)	17 (19.8%)	20 (18.0%)
Number of questionnaire respondents excluding young or attending surgeons	42 (33.3%)	35 (56.5%)	26 (37.7%)	33 (47.8%)	44 (48.4%)
Medical students	16 (38.1%)	12 (34.3%)	5 (19.2%)	12 (36.4%)	14 (31.8%)
Junior residents (postgraduate years 1-2)	22 (52.4%)	21 (60.0%)	17 (65.4%)	21 (63.6%)	25 (56.8%)
Senior residents (postgraduate years 3-5)	4 (9.5%)	2 (5.7%)	4 (15.4%)	0 (0.0%)	5 (11.4%)

### Residency entry data

**Fig. 2:**
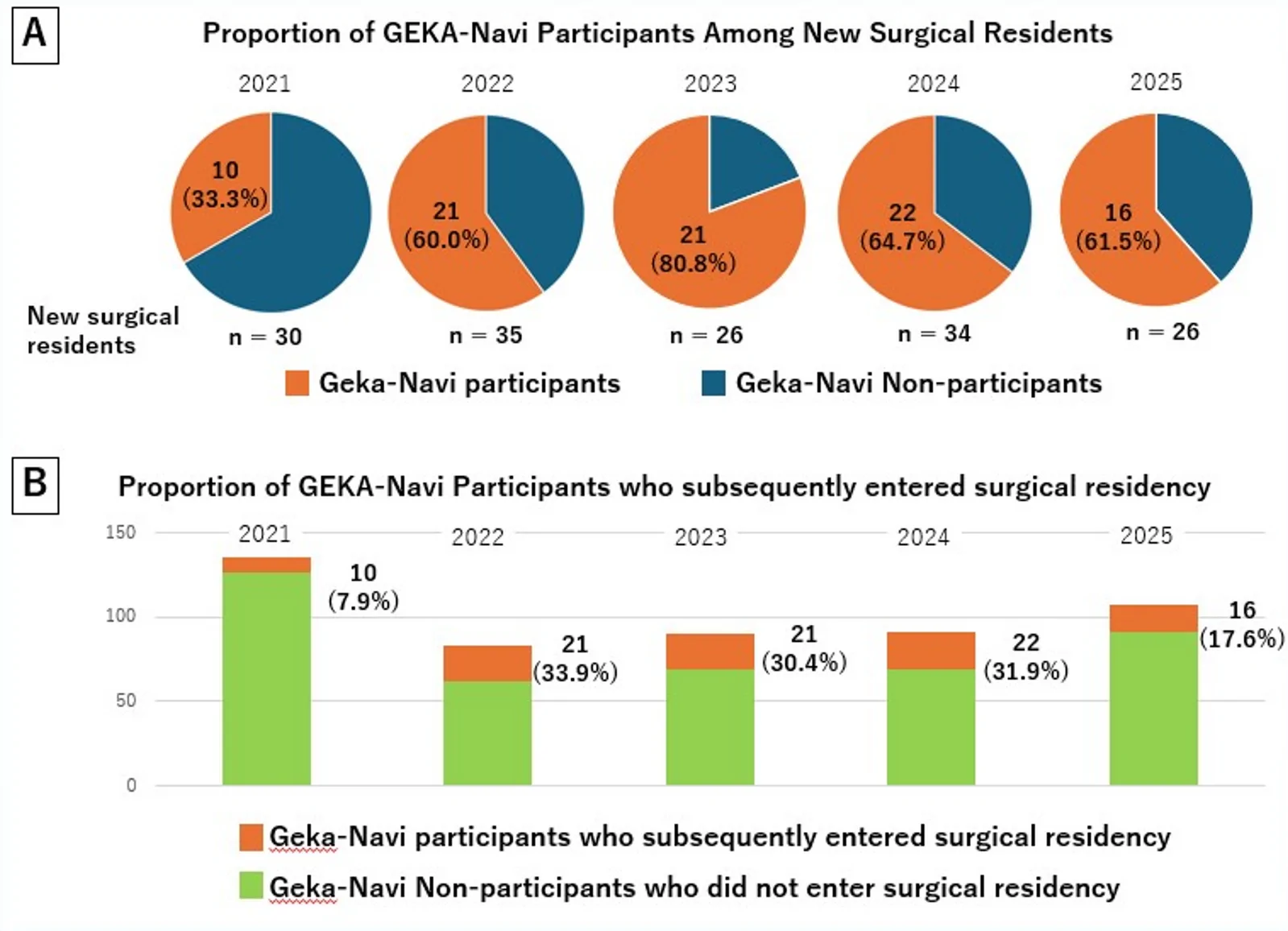
Association between GEKA-Navi participation and surgical residency entry (2021–2025). (**A**) Proportion of new surgical residents who had previously participated in GEKA-Navi from 2021 to 2025. Overall, 59.6% (90 of 151) of residents had attended GEKA-Navi before entering surgical residency. (**B**) Proportion of GEKA-Navi participants who subsequently entered surgical residency programs each year. The proportion ranged from 7.9% to 33.9% during the study period

A total of 151 residents entered eight participating surgical departments between 2021 and 2025. Among them, 90 (59.6%) had previously participated in the GEKA-Navi program. Figure 2A illustrates the annual proportion of residency entrants with and without prior GEKA-Navi participation. Furthermore, we descriptively examined the proportion of GEKA-Navi participants who subsequently entered surgical residency programs at our institution, ranging from 7.9% to 33.9% across the study period (Fig. 2B).

### Questionnaire-based participant evaluations

#### Satisfaction with the Program

**Fig. 3:**
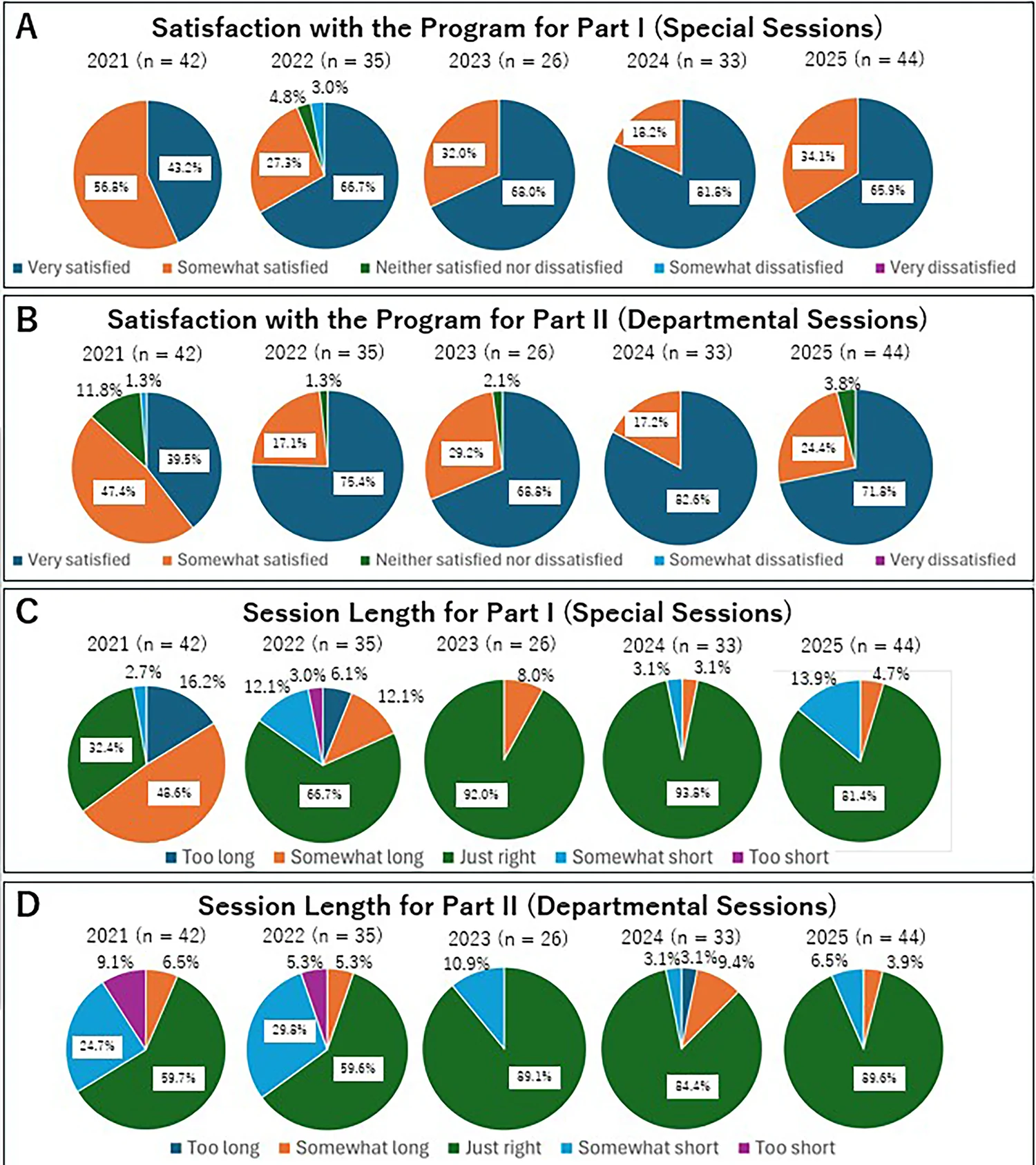

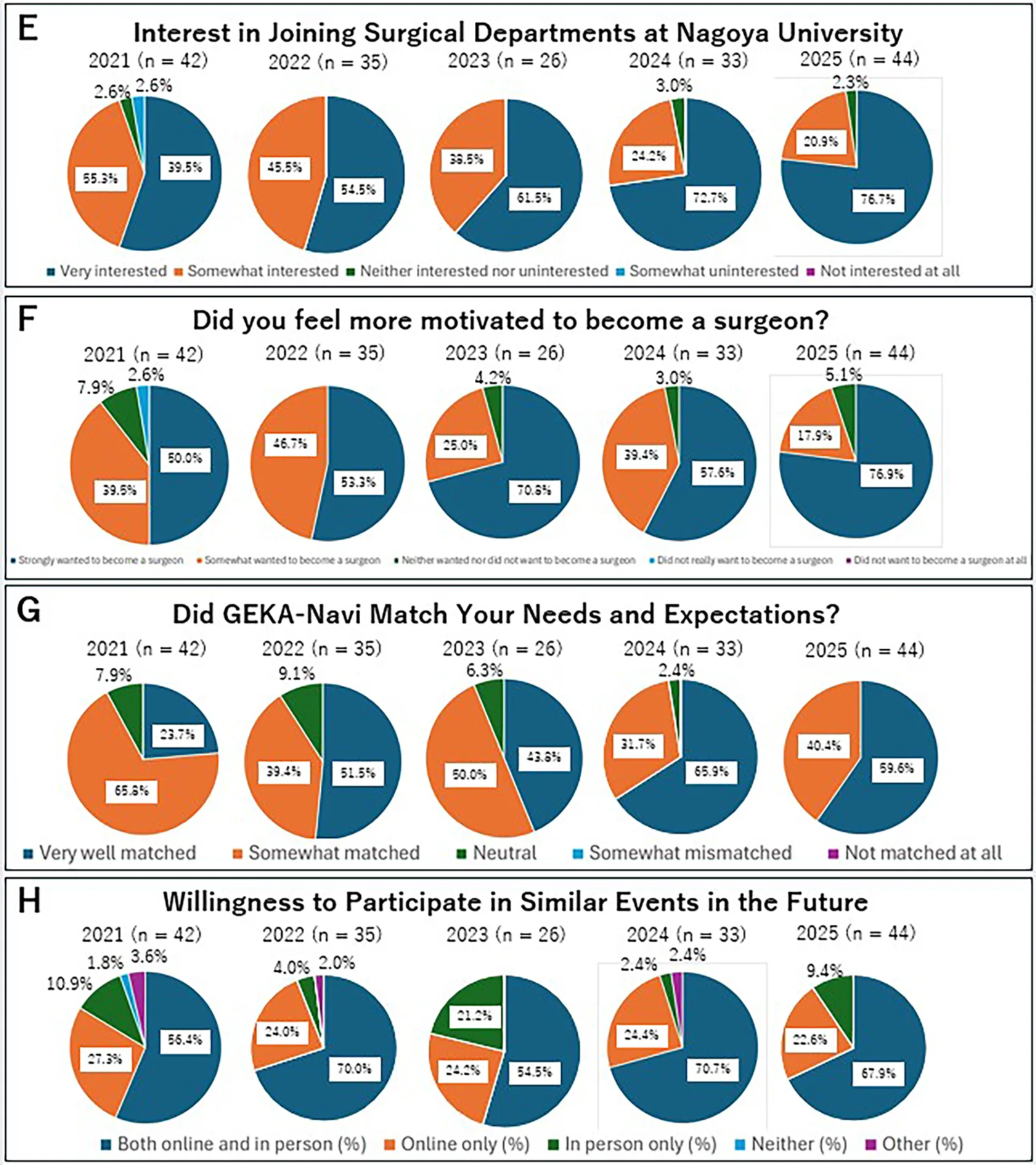
Participant evaluations of GEKA-Navi from 2021 to 2025. (**A**) Satisfaction with the program for part I (Special sessions), (**B**) Satisfaction with the program for part II (Departmental sessions), (**C**) Session length for Part I (Special sessions), (**D**) Session length for Part II (Departmental sessions). (**E**) Interest in joining surgical departments at Nagoya University, (**F**) Did you feel motivated to become a surgeon? (**G**) Degree to which GEKA-Navi matched participants’ needs and expectations. (**H**) Willingness to participate in future GEKA-Navi events (online and/or in-person formats)

The 2025 survey found that 98% of participants reported that the event increased their interest in the surgical departments at Nagoya University (Fig. 3A and B). Regarding overall satisfaction:

- For Part I (special sessions), 66% answered “very satisfied” and 34% “somewhat satisfied.”
- For Part II (departmental sessions), 74% were “very satisfied” and 25% were “somewhat satisfied.”

Over the 5 years, satisfaction scores consistently remained above 90% (combined “very” and “somewhat” satisfied).

### Session length

In the first year of GEKA-Navi (2021), responses regarding the duration of Part I revealed that a substantial proportion of participants perceived the session as “somewhat long.” However, over the subsequent years, the proportion of participants who considered the duration to be “just right” gradually increased. In recent years, over 80% of participants have reported that the duration of Part I was appropriate. Responses during the first 2 years for the departmental breakout sessions (Part II) indicated that the sessions were “somewhat short,” reflecting the desire of participants for more in-depth discussions with each surgical department. In response, the duration of Part II was extended by approximately 1.5-fold starting in 2023 (the third year of the program). After this modification, over 80% of participants evaluated the session length as “just right.” Fig. 3C and D illustrate these trends in detail.

### Perceived program value and impact on motivation

Several consistent trends were observed over time when participants were asked whether the event helped them consider a career in surgery. In the earlier years, responses regarding interest in joining surgical departments at Nagoya University were more frequently categorized as “somewhat interested.” However, the proportion of participants who selected “very interested” gradually increased over subsequent years, indicating a strengthening of institutional interest among attendees (Fig. 3E). The combined proportion of participants who answered “strongly wanted to become a surgeon” or “somewhat wanted to become a surgeon” to the question “Did you feel more motivated to become a surgeon?” exceeded 80% in every year of the study period. Moreover, the percentage of participants selecting “strongly wanted to become a surgeon” progressively increased over time, indicating a growing depth of motivation (Fig. 3F). Whether GEKA-Navi matched participants’ needs and expectations, approximately 90% or more of participants across all years answered, “very well matched” or “somewhat matched.” Notably, the proportion of “very well matched” responses increased year by year (Fig. 3G). Concerning the question regarding willingness to participate in similar events in the future, over 90% of participants each year expressed interest in attending future iterations of GEKA-Navi in some format—whether online, in person, or both (Fig. 3H).

## Discussion

In this study, we investigated the effectiveness of GEKA-Navi, an eight-department, multi-surgical online recruitment event held annually at Nagoya University Hospital between 2021 and 2025. Participant satisfaction and interest in surgical careers remained consistently high throughout the study period, with approximately 98% of the participants expressing an increased interest in joining the surgical department at our institution. Furthermore, actual residency entry data analysis revealed that approximately 60% of newly recruited surgical residents had previously participated in GEKA-Navi, indicating that the event provided descriptive insights into participant perceptions and recruitment patterns. The novelty of this study lies in its comprehensive assessment of a multidepartment online surgical recruitment initiative sustained over 5 years, integrating both longitudinal questionnaire data and objective residency entry outcomes.

The shortage of surgeons in Japan has become a critical societal concern, and similar trends have been reported in North America and Europe. Survey studies conducted among US medical students have identified several factors that contribute to the decline in interest in surgical careers, including long working hours, difficulty in maintaining work–life balance, the scarcity of female surgical role models, and limited access to mentorship [4, 8, 9]. Conversely, the technical appeal of surgery and the significant effect of surgical treatment on patient outcomes have been recognized to positively influence career interest [10–12]. Notably, a consistent finding across such studies is the importance of early exposure to surgical experiences as a means of cultivating interest in the field [5, 6, 13]. Intentionally designing opportunities for medical students to engage with surgical careers at an early stage is considered a globally valid approach to addressing the shortage of surgeons [3, 8]. Our program, GEKA-Navi, provides comparable multispecialty surgical early exposure, allowing participants to gain a panoramic understanding of the diversity and appeal of surgical fields. A distinctive characteristic of GEKA-Navi is the inclusion of not only senior faculty, but also younger surgeons who serve as instructors and relatable role models. This generational proximity enables authentic communication of real-world surgical career trajectories. These characteristics distinguish GEKA-Navi from conventional unidirectional career sessions and may contribute meaningfully to improving participants’ engagement and motivation toward surgical careers. This multilayered and integrated design may represent a distinct approach compared with conventional department-based recruitment strategies.

One of the major strengths of GEKA-Navi is its structured design, which promotes early exposure to surgical careers and particularly targets medical students and junior residents. Additional distinguishing features include an integrated, cross-disciplinary approach involving eight surgical departments and the ability to visualize and track positive shifts in participants’ attitudes after the event. The online format further contributed to its practicality by reducing the organizational burden on surgical faculty and related costs, while facilitating participation from geographically distant trainees. This likely enabled outreach to students and residents who might otherwise have been unable to attend an in-person session, thereby highlighting the potential of online platforms as effective recruitment tools in the post-pandemic era of research. Moreover, GEKA-Navi serves as a gateway for deeper engagement in surgical training. For example, participants were frequently encouraged to participate in subsequent opportunities, including departmental observerships, monthly hands-on training sessions, and cadaver surgical training programs held twice a year in the Department of Thoracic Surgery. These follow-up pathways enable a stepwise and structured recruitment process that commences with GEKA-Navi and culminates in immersive surgical experiences [14].

Survey-based research has proven to be a valuable tool for understanding the motivations and barriers associated with pursuing a surgical career [1, 2, 15]. The high levels of satisfaction and motivational impact observed in the GEKA-Navi questionnaire responses indicate that the program may help address several known barriers, including a lack of information, unclear career trajectories, and limited access to mentors, by effectively communicating the appeal of surgery and providing a more tangible vision of future surgical careers. The decision to pursue surgery is frequently shaped by both positive motivators (e.g., technical appeal and sense of accomplishment) and deterrents (e.g., concerns about work–life balance, training environment, and long-term career uncertainty) [4]. Therefore, addressing the ongoing shortage of surgeons requires a dual-pronged strategy: establishing high-quality training environments and ensuring that their value is communicated accurately and early in medical education. Hence, GEKA-Navi serves as an intervention that fulfills the latter role, making the appeal of surgical careers visible and accessible at an early age.

Questionnaire data analysis revealed that all evaluated domains demonstrated a gradual numerical improvement over time, including participant satisfaction and interest in surgical careers. Alongside this trend, motivation to pursue surgical training or to consider a surgical career strengthened year by year, potentially affected by the continued refinement of the GEKA-Navi program itself. These findings indicate that effective recruitment of individuals with latent potential to become surgeons requires forward-looking approaches that are carefully aligned with the perspectives and values of each new generation. The organizers of such recruitment initiatives frequently belong to different generations than the trainees they seek to engage, and fully understanding the expectations and concerns of younger cohorts from an external viewpoint is inherently limited. Hence, continuously soliciting structured feedback through participant questionnaires and implementing iterative program improvements are key components of sustainable recruitment strategies. Our results indicate that such an approach may help increase engagement and eventual entry into surgical training. Importantly, the results of this study reveal that the observed attitudinal changes among participants were not only transient expressions of satisfaction, but may also translate into tangible behavioral changes. A substantial proportion of newly recruited surgical residents had previously participated in GEKA-Navi, thereby supporting this interpretation. The proportion of GEKA-Navi participants who subsequently entered surgical residency programs differed across years, reflecting fluctuations in participants’ stage of training, cohort size, and external factors, rather than the effect of the program alone. Further, we analyzed the proportion of GEKA-Navi participants who subsequently entered surgical residency programs. Approximately one-third of participants ultimately joined surgical departments at our institution. We investigated the number of newly recruited surgical residents before and after GEKA-Navi implementation, with 177 residents in the pre-implementation period (2016–2020) and 151 in the post-implementation period (2021–2025). However, external factors (e.g., COVID-19 pandemic), which may have affected recruitment trends, influenced these periods. Therefore, a simple pre–post comparison is not appropriate for evaluating the impact of the program. Accordingly, the findings of this study should not be interpreted as demonstrating a causal effect on overall recruitment volume, but rather as providing descriptive insights into the association between GEKA-Navi participation and subsequent residency entry. Continued refinement and evaluation of the GEKA-Navi program may help inform future recruitment strategies.

This study has several important limitations. First, not all GEKA-Navi participants completed the post-event questionnaire, which raises the possibility of some response bias, as individuals with more favorable impressions may have been more likely to respond [16]. Second, this study did not include a comparison group, and baseline data regarding participants’ pre-existing interest in surgical careers were not available. Therefore, directly comparing participants and non-participants or adjusting for baseline differences in career motivation was not possible. Changes in participants’ motivation before and after the intervention could not be quantitatively assessed. Furthermore, some selection bias is likely to be present because GEKA-Navi primarily targeted individuals with an existing interest in surgical careers. Participants may have been inherently more inclined toward surgical training, which could have affected their participation in the program and subsequent career choices. Furthermore, this study was retrospective and observational in nature, and the evaluated outcomes primarily represented short-term indicators, including satisfaction and self-reported motivation. Moreover, the study population included participants at different stages of training, including medical students, junior residents, and senior ones. These groups differed in their career decision-making stages; thus, this heterogeneity may have affected the interpretation of questionnaire responses and recruitment-related outcomes. The observation that a substantial proportion of newly recruited surgical residents had previously participated in GEKA-Navi provides an objective indicator of a potential association; however, the study design does not allow for causal inference regarding the effect of the program on recruitment outcomes. Finally, the long-term outcomes, including sustained entry into surgical training, retention within the surgical workforce, and long-term career persistence, were not evaluated and should be investigated in future studies.

## Conclusion

Our recruitment event for surgical residency programs was associated with high participant satisfaction and increased interest in surgical careers. This study provides descriptive insights into participants’ perceptions and recruitment patterns within a structured multidepartmental recruitment initiative. Recruiting individuals who can pursue surgical careers requires ongoing adaptation to generational values, expectations, and learning styles. However, mid-career faculty members frequently conduct such programs. Therefore, an inherent gap may exist between the organizers and the participants. Therefore, continuous evaluation through participant feedback is essential. Iterative refinement based on such feedback may contribute to sustainable growth in future surgical workforce.

## Data Availability

The datasets used in the present study are available from the corresponding author upon reasonable request.

## Abbreviations

GEKA-Navi: Multi-Surgical Department Online Recruitment Event (“GEKA Navigation”)
PGY: Postgraduate Year
COVID-19: Coronavirus Disease 2019
